# Impact of thyroid autoimmunity on the cumulative live birth rates after IVF/ICSI treatment cycles

**DOI:** 10.1186/s12884-024-06411-4

**Published:** 2024-04-02

**Authors:** Ning Huang, Lixue Chen, Zhiqiang Yan, Hongbin Chi, Jie Qiao

**Affiliations:** 1 Center for reproductive medicine, Department of Obstetrics and Gynecology, Peking University Third Hospital, 49 North Garden Rd, Beijing, China; 2 National Clinical Research Center for Obstetrics and Gynecology, Peking University Third Hospital, Beijing, China; 3 Key Laboratory of Assisted Reproduction, Peking University, Ministry of Education, Beijing, China; 4 Beijing Key Laboratory of Reproductive Endocrinology and Assisted Reproductive Technology, Beijing, China; 5 Beijing Advanced Innovation Center for Genomics, Beijing, China; 6 Peking-Tsinghua Center for Life Sciences, Peking University, Beijing, China

**Keywords:** Cumulative live birth rate, Thyroid autoimmunity, Infertility, In vitro fertilization

## Abstract

**Background:**

Cumulative live birth rate (CLBR) is considered as the most important endpoint for assessing the probability of having a baby in a complete in vitro fertilization/intracytoplasmic sperm injection (IVF/ICSI) treatment cycle. Many previous studies have focused on the association between thyroid autoimmunity (TAI) and live birth rate after first embryo transfer cycle, however, evidence on whether the presence of TAI affects the CLBR is lacking. The purpose of this study is to investigate the impact of TAI on the CLBR in a complete IVF/ICSI cycle.

**Methods:**

This retrospective study included 12,796 women who underwent their first IVF/ICSI treatment between January 2019 and February 2021. Based on the levels of thyroid antibodies, 2,603 women were assigned to the TAI group, and 10,193 women were assigned to the control group. Subgroup analysis was performed according to the different causes of infertility (including male factor only, ovulation disorder, tubal factor, endometriosis and unexplained infertility) and different types and titres of thyroid antibodies. The primary outcome in this study was CLBR, which included live births from the fresh embryo transfer cycle and all subsequent frozen-thawed embryo transfer cycles performed before December 2021.

**Results:**

There was no significant difference in the CLBR between the TAI and control groups, even after adjusting for relevant confounders including age, body mass index, cause of infertility, thyroid function, protocols of controlled ovarian stimulation, type of transfer (fresh vs. frozen), type of transferred embryo (cleavage-stage embryo vs. blastocyst), and fertilization method (IVF vs. ICSI) (cumulative live birth: 50.6% vs. 52.1%, OR 0.94, 95% CI 0.86–1.02, adjusted OR 0.97, 95%CI 0.89–1.06). Subgroup analysis showed that no significant difference was observed in CLBR between the TAI and control groups for all causes of infertility, except for infertility attributed to endometriosis. Among women with endometriosis, the CLBR was significantly lower in the TAI group than that in the control group; however, this difference was not significant after adjusting for potential confounders including age, body mass index, thyroid function, protocols of controlled ovarian stimulation, type of transfer (fresh vs. frozen), type of transferred embryo (cleavage-stage embryo vs. blastocyst), and fertilization method (IVF vs. ICSI) (cumulative live births: 43.1% vs. 51.0%, OR 0.73, 95% CI 0.53–0.99, adjusted OR 0.74, 95% CI 0.53–1.02). Another subgroup analysis demonstrated that the type and titre of thyroid antibody did not affect CLBR in women with TAI.

**Conclusions:**

In our study, there was no significant difference in the CLBR between women with TAI and those without TAI, which suggests that TAI did not affect the chances of having a baby in a complete IVF/ICSI treatment cycle.

## Background

Thyroid autoimmunity (TAI) is defined as the presence of circulating antibodies against thyroid peroxidase or thyroglobulin. Compared with fertile women, a higher prevalence of TAI has been reported in women with infertility, especially in those with polycystic ovarian syndrome (PCOS) and endometriosis [1, 2]. Moreover, infertile women with TAI may be prone to adverse assisted reproductive outcomes, including decreased number of retrieved oocytes, decreased rates of live birth and increased rates of miscarriage [3–5]. The 2021 European Thyroid Association guidelines recommend screening for thyroid antibodies in women with infertility before seeking medical treatment [6].

The association between TAI and assisted reproductive outcomes after first embryo transfer cycle has been studied for several years. A meta-analysis that reviewed 12 studies published between 1990 and 2015 demonstrated that TAI was associated with an increased risk of miscarriage and a decreased rate of live birth [3]. However, several recent cohort studies reported inconsistent results, revealing no significant difference in miscarriage and live birth rates between women with and without TAI [7–9]. These inconsistent results may result from the cross-sectional design, which aims to measure the impact of TAI on assisted reproductive outcomes after the first embryo transfer cycle and is prone to be affected by the different types of transfer (fresh or frozen-thawed cycle), different types of transferred embryos (cleavage-stage embryos or blastocysts), embryo quality, and patient condition in the first transferred cycle.

Compared with the live birth rate after the first transfer cycle, cumulative live birth rate (CLBR) is a more stable outcome in assisted reproductive technology, which evaluates the possibility of having a baby from the first and all subsequent frozen-thawed transfer cycles and reflects the quality of all embryos obtained from a complete IVF/ICSI treatment cycle. To our knowledge, only one previous study that included 2406 infertile patients investigated the impact of TAI on CLBR, however, this study defined TAI as thyroid peroxidase antibody (TPOAb) positivity, and women with thyroglobulin antibody (TgAb) positivity were neglected. In addition, this study did not adjust the impact of the causes of infertility, which may be an important potential confounder [10].

Therefore, we conducted this large-scale retrospective study to investigate the impact of TAI on CLBR in women undergoing IVF/ICSI treatment. Subgroup analysis was performed to further assess the association between TAI and CLBR in women with different causes of infertility. Among the women with TAI, we further investigated the role of different types and titres of thyroid antibodies in CLBR.

## Methods

### Patients

**Fig. 1:**
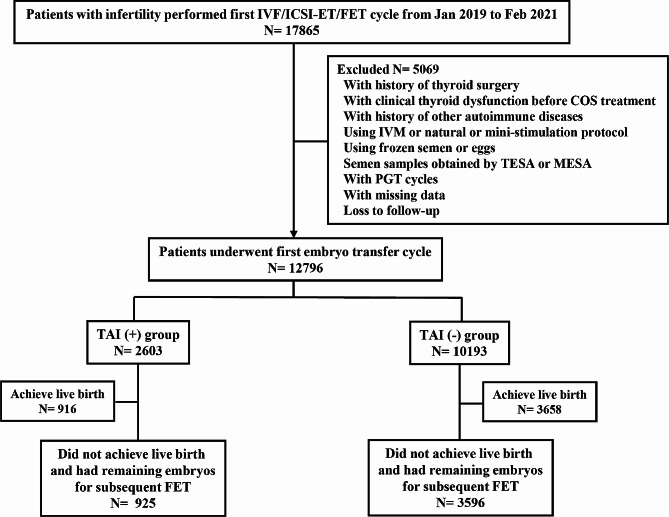
Flowchart of study cohort selection and follow-up procedures Abbreviations: IVF, in vitro fertilization; ICSI, intracytoplasmic sperm injection; TESA, testicular sperm aspiration; MESA, microsurgical epididymal sperm aspiration; PGT, preimplantation genetic testing; TAI, thyroid autoimmunity; ET, embryo transfer; FET, frozen embryo transfer

This retrospective cohort study was conducted at the Reproductive Center of Peking University Third Hospital between January 2019 and February 2021. The study was approved by the Peking University Third Hospital Medical Science Research Ethics Committee. A total of 17,865 women with infertility underwent their first IVF/ICSI treatment, obtained viable embryos and subsequently underwent embryo transfer cycle. Women were not eligible if they had a history of thyroid surgery or clinical thyroid dysfunction prior to controlled ovarian stimulation (COS) treatment, had a history of other autoimmune diseases, underwent in vitro maturation, natural or mini-stimulation protocols, used frozen semen or eggs, obtained semen samples by testicular sperm aspiration or microsurgical epididymal sperm aspiration, or if they underwent pre-implantation genetic testing (Fig. 1).

Finally, we included 12,796 women in our study. Based on the TgAb and/or TPOAb levels, 2,603 women were included in the TAI group, while 10,193 women were included in the control group. All women involved were followed up until December 31, 2021, and the follow-up was terminated when a live birth was achieved or when all embryos obtained from the first oocyte retrieval had been used. Cycles up to and including the first live birth were included, whereas cycles after the first live birth were excluded.

### Assisted reproductive technology procedures

All patients received a COS regimen. Gonadotropin-releasing hormone (GnRH) agonist was used to achieve pituitary inhibition in women treated using ultralong-term or long-term protocols. Recombinant gonadotropins were initiated once down-regulation was achieved. In patients treated with the antagonist protocol, recombinant gonadotropins were initiated on day 2 of the menstrual cycle, and a GnRH antagonist was administered when at least one follicle reached 12 mm in diameter until the day of the trigger. When at least two follicles reached 18 mm, 250 mg recombinant human chorionic gonadotropin (HCG) (Eiser, Serono, Germany) was administered to trigger oocyte maturation and oocyte retrieval was performed by transvaginal needle aspiration 34–36 h after HCG administration. The retrieved oocytes were subsequently inseminated by conventional IVF or ICSI. Normal fertilization was defined as the presence of two pronuclei, typically assessed approximately 16–18 h after insemination. Embryos were evaluated 3 days after fertilization according to the quality, number, size of the blastomeres, and the amount of anucleate fragmentation. Fresh or frozen-thawed embryo transfer was decided according to the condition of the patients, and up to two day 3 embryos or blastocysts were transferred. In the frozen-thawed embryo transfer cycle, the endometrium was prepared using a natural or hormone replacement protocols. For patients undergoing a natural protocol, follicular growth was monitored using transvaginal ultrasound from days 8 to 10 of the menstrual cycle until ovulation. Frozen-thawed embryo transfer was scheduled 3 days after ovulation for cleavage-stage embryos or 5 days after ovulation for blastocysts. For patients undergoing a hormone replacement protocol, oral oestradiol valerate (Progynova; Bayer Schering Pharma AG) at a daily dose of 6 mg was given on days 2–3 of their menstrual cycle. Subsequently, a transvaginal ultrasound was performed after 10 days to monitor the endometrial thickness. Progesterone treatment was initiated when the endometrial thickness reached 8 mm. Freeze-thawed embryo transfer was scheduled 5 days after using progesterone for cleavage-stage embryos or 7 days after using progesterone for blastocysts.

### Baseline characteristics and laboratory testing

The baseline characteristics of the included women were collected before the COS treatment, including age, body mass index (BMI), duration of infertility, and type of infertility (primary or secondary). The causes of infertility were assessed and broadly classified into five categories: male factors only, tubal factors, ovulatory disorders, endometriosis, and unexplained infertility. Ovarian reserve was assessed based on the levels of anti-Mullerian hormone (AMH) and basal female hormones, including follicle-stimulating hormone, luteinizing hormone (LH), and oestradiol, which were measured from the 2nd to 4th day of an unstimulated menstrual cycle. All patients were tested for thyroid function before initiating COS treatment. Parameters relevant to thyroid function, including serum thyroid-stimulating hormone (TSH), free thyroxine (FT4), TPOAb, and TgAb levels, were measured using a fully automatic chemiluminescence immunoassay analyzer (ADVIA Centaur XP, Siemens Healthcare Diagnostics). The reference values were 0.55–4.78 uIU/mL for TSH and 0.89–1.80 ng/dL for FT4. Concentrations > 60 IU/mL were considered positive for TPOAb and TgAb. Blood samples for LH, oestradiol, and progesterone were collected on the day of the trigger for the female hormone testing. Sperm concentration and progressive motility were assessed before COS treatment using computer-assisted semen analysis following the fifth edition of the World Health Organization laboratory standards for human semen and sperm.

### Study outcomes

The primary outcome in this study was CLBR, which included live births from the first transfer cycle and all subsequent frozen-thawed cycles performed before December 2021. Live birth was defined as the delivery of at least one survived newborn, irrespective of the gestation duration.

### Statistical analysis

Continuous variables with normal distribution were presented as mean (standard deviation [SD]), and when the parameters were not normally distributed, the median (interquartile range) was reported. Statistical comparisons of continuous data were performed using the t-test or Mann–Whitney U test. Data were expressed as the number of cases (percentage) for categorical variables. Chi-square tests were used to compare differences in categorical variables. Multivariate logistic regression analysis was used to adjust for relevant factors, including age, body mass index, cause of infertility, FT4, TSH, COS protocols, type of transfer (fresh vs. frozen), type of transferred embryo (cleavage-stage embryo vs. blastocyst), and fertilization method (IVF vs. ICSI). Odds ratios (ORs) with 95% confidence intervals (CIs) were calculated and presented. A two-sided *P*-value < 0.05 was considered statistically significant. All analyses were performed using the SPSS 24 statistical software.

## Results

The baseline characteristics of patients were presented in Table 1. A total of 12,796 women were screened in our study, including 2,603 women with TAI and 10,193 women without TAI. No significant differences were observed in BMI or type of infertility. The characteristics relevant to the ovarian reserve, including the levels of basal female hormones and AMH, were similar between the two groups. However, the mean age of the women with TAI was significantly higher than that of the control group (mean [SD]:32.7 [4.1] vs. 32.4 [4.3], *p* = 0.001). A significant difference was also observed regarding the causes of infertility (*p* = 0.028). The prevalence of male factors was higher, and the percentage of tubal factors was lower in women with TAI than in the control group. However, sperm concentration and motility between the two groups were similar. A significant difference was observed in the thyroid function between the TAI and control groups. A significantly lower level of FT4 (median [interquartile range]:1.3 [1.2–1.4] vs. 1.3 [1.2–1.4], *p*<0.001), and a significantly higher level of TSH was observed (median [interquartile range]:2.2 [1.5–3.0] vs. 2.0 [1.4–2.7], *p*<0.001) in women with TAI than in controls.

**Table 1:** Baseline characteristics of patients

Characteristics	TAI group (*N* = 2603)	Control group (*N* = 10,193)	*P*-value
Age, mean (SD), years	32.7 (4.1)	32.4 (4.3)	0.001
BMI, mean (SD), kg/m^2^	22.9 (3.7)	22.8 (3.7)	0.094
Duration of infertility, median (IQR), years	3.0 (2.0–4.0)	3.0 (2.0–4.0)	0.832
Type of infertility, No. (%)			
Primary	1482 (56.9)	5894 (57.8)	0.412
Secondary	1121 (43.1)	4299 (42.2)	
Cause of infertility, No. (%) ^a^			
Male factor only	771 (29.6)	2761 (27.1)	0.028
Ovulation disorder	429 (16.5)	1623 (15.9)	
Tubal factor	891 (34.2)	3768 (37.0)	
Endometriosis	197 (7.6)	842 (8.3)	
Unexplained	315 (12.1)	1199 (11.8)	
Basal FSH, median (IQR), mIU/mL ^b^	6.4 (5.1–7.9)	6.3 (5.0-7.8)	0.581
Basal LH, median (IQR), mIU/mL ^b^	3.5 (2.3–5.2)	3.6 (2.3–5.2)	0.319
Basal oestradiol, median (IQR), pmol/L ^b^	152.0 (110.0-195.0)	152.0 (110.0-196.0)	0.917
AMH, median (IQR), ng/mL	2.6 (1.4–4.4)	2.5 (1.4–4.4)	0.317
FT4, median (IQR), ng/dL	1.3 (1.2–1.4)	1.3 (1.2–1.4)	< 0.001
TSH, median (IQR), mIU/L	2.2 (1.5-3.0)	2.0 (1.4–2.7)	< 0.001
Sperm concentration, median (IQR), million/mL	49.7 (24.9–85.2)	52.0 (26.5–86.6)	0.102
Sperm motility, median (IQR), %	28.0 (15.7–43.5)	29.0 (16.2–44.0)	0.227

BMI, body mass index; FSH, follicle-stimulating hormone; LH, luteinizing hormone; AMH, anti-Müllerian hormone; FT4, free thyroxine; TSH, thyroid-stimulating hormone; COS, controlled ovarian stimulation; IQR, interquartile range; SD, standard deviation

^a^ Cause of infertility is the most important indication for couples who undergo IVF/ICSI treatment

^b^ Testing for basal FSH, LH, and oestradiol was performed between day 2 and day 4 of the menstrual cycle

As shown in Table 2, no significant differences were observed regarding the protocols for COS, duration of stimulation, or total dose of gonadotropin. On the day of trigger, oestradiol level was significantly higher in women with TAI (median [interquartile range]: 7986.0 [5191.0–12032.0] vs. 7637.5 [5037.0–11831.3], *p* = 0.015), while the levels of LH and progesterone were similar between the two groups. No significant difference was observed in the number of retrieved oocytes between women with TAI and those without TAI. The percentage of women fertilized using conventional IVF or ICSI significantly differed between the two groups (IVF: 74.3 vs. 76.3; ICSI: 25.7 vs. 23.7, *p* = 0.034). However, no significant difference was observed in fertilization rate or number of good-quality embryos between the two groups.

**Table 2:** Protocols for controlled ovarian stimulation and data on IVF and first embryo transfer cycle

Characteristics	TAI group *N* = 2603	Control group *N* = 10,193	*P*-value
Protocols for COS, No. (%)			
Ultralong GnRH agonist	296 (11.4)	1142 (11.2)	0.304
Long GnRH agonist	458 (17.6)	1928 (18.9)	
GnRH antagonist	1849 (71.0)	7123 (69.9)	
Duration of stimulation, median (IQR), days	11.0 (9.0–12.0)	11.0 (9.0–12.0)	0.683
Total dose of gonadotropin, median (IQR), IU	2437.5 (1800.0-3300.0)	2475.0 (1800.0-3275.0)	0.929
LH level on day of trigger, median (IQR), mIU/mL	1.2 (0.6–2.3)	1.2 (0.6–2.5)	0.176
Oestradiol level on day of trigger, median (IQR), mIU/mL	7986.0 (5191.0-12032.0)	7637.5 (5037.0-11831.3)	0.015
Progesterone level on day of trigger, median (IQR), mIU/mL	2.0 (1.4-3.0)	2.0 (1.4-3.0)	0.545
Number of oocytes retrieved, median (IQR)	11.0 (7.0–17.0)	11.0 (7.0–17.0)	0.718
Fertilization per oocyte inseminated or injected, median (IQR), % ^a^	0.7 (0.5–0.8)	0.7 (0.5–0.8)	0.967
Fertilization No. (%)			
IVF	1933 (74.3)	7773 (76.3)	0.034
ICSI	670 (25.7)	2420 (23.7)	
Number of good-quality embryos, median (IQR) ^b^	5.0 (2.0–8.0)	5.0 (2.0–8.0)	0.812

GnRH: gonadotropin-releasing hormone; LH: luteinizing hormone; COS: controlled ovarian stimulation; IQR: interquartile range; SD: standard deviation

^a^ Normal fertilization was defined as the number of zygotes with two pronuclei. In the IVF group, the denominator was the number of retrieved oocytes. In the ICSI group, the number of metaphase II oocytes was the denominator

^b^ Embryos were evaluated on the third day after fertilization. Good-quality embryos were developed from two-pronuclei zygotes and met the following criteria: (1) more than five blastomeres, (2) size difference of less than 20%, and (3) fragmentation of less than 50%

**Fig. 2:**
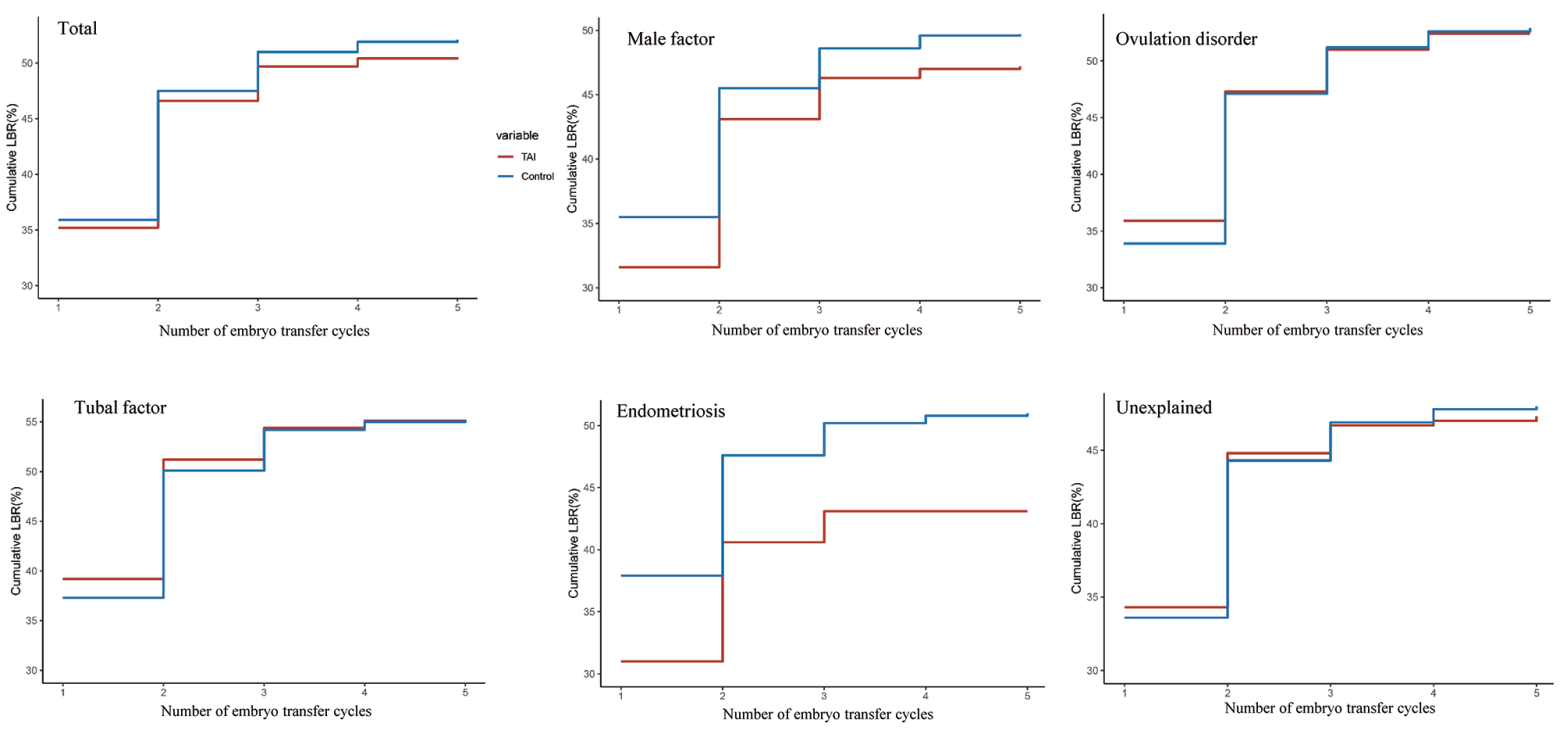
Observed cumulative live birth rates among different groups

This study included 2,603 women with TAI and 10,193 women without TAI, resulting in 3,900 and 15,303 embryo transfer cycles with 1,316 and 5,316 live births, respectively. There was no significant difference in the CLBR between the TAI and control groups, even after adjusting for relevant confounders (cumulative live birth: 50.6% vs. 52.1%, OR 0.94, 95% CI 0.86–1.02, adjusted OR 0.97, 95%CI 0.89–1.06). Subgroup analysis was conducted to evaluate CLBR according to the cause of infertility. No significant difference in CLBR was observed between the two groups for all causes of infertility except for infertility attributed to endometriosis. Among women with endometriosis, the cumulative likelihood of live births was significantly lower in the TAI group than in the control group; however, the difference was not significant after adjusting for potential confounders (cumulative live births: 43.1% vs. 51.0%, OR 0.73, 95% CI 0.53–0.99, adjusted OR 0.74, 95% CI 0.53–1.02) (Table 3; Fig. 2).

**Table 3:** Comparison of cumulative live birth outcomes between TAI and control groups

Group	TAI group			Control group			OR (95% CI)	Adjusted OR (95% CI)
	No. of patients	No. of LB	CLBR (%) ^a^	No. of patients	No. of LB	CLBR (%)		
Total	2603	1316	50.6	10,193	5316	52.1	0.94 (0.86–1.02)	0.97 (0.89–1.06) ^*^
Male factor only	771	364	47.2	2761	1372	49.7	0.91 (0.77–1.06)	0.93 (0.78–1.09) ^#^
Ovulation disorder	429	225	52.4	1623	859	52.9	0.98 (0.79–1.21)	1.03 (0.82–1.29) ^#^
Tubal factor	891	492	55.2	3768	2081	55.2	1.00 (0.86–1.16)	1.03 (0.88–1.20) ^#^
Endometriosis	197	85	43.1	842	429	51.0	0.73 (0.53–0.99)	0.74 (0.53–1.02) ^#^
Unexplained	315	150	47.6	1199	575	48.0	0.99 (0.77–1.27)	1.03 (0.79–1.36) ^#^

LB, live birth; CLBR, cumulative live birth rate; OR, odds ratio

^a^ CLBR: cumulative live birth was defined as the delivery of one or more living infants in the first and subsequent frozen-thawed cycles. The cumulative live birth rate was calculated as the number of cumulative live births divided by the number of COS cycles

^*^ The multivariate model was adjusted for age, body mass index, cause of infertility, FT4, TSH, COS protocols, type of transfer (fresh vs. frozen), type of transferred embryo (cleavage-stage embryo vs. blastocyst), and fertilization method (IVF vs. ICSI).

^#^The multivariate model was adjusted for age, body mass index, FT4, TSH, COS protocols, type of transfer (fresh vs. frozen), type of transferred embryo (cleavage-stage embryo vs. blastocyst), and fertilization method (IVF vs. ICSI).

Based on the types of thyroid antibodies, the women with TAI were divided into three subgroups: co-positive for TgAb and TPOAb, isolated positive for TgAb, and isolated positive for TPOAb. Subgroup analysis did not reveal significant differences in the rates of cumulative live births among these three groups compared with women without TAI (Table 4).

**Table 4:** Subgroup analysis of cumulative live birth outcomes according to different types of thyroid antibodies

Group	No. of patients	No. of LB	CLBR (%) ^a^	OR (95%CI)	Adjusted OR (95%) ^*^
Co-positive for TGAb and TPOAb	1244	630	50.6	0.94 (0.84–1.06)	0.99 (0.87–1.12)
Isolated positive for TGAb	779	400	51.3	0.97 (0.84–1.12)	0.98 (0.84–1.14)
Isolated positive for TPOAb	580	286	49.3	0.89 (0.76–1.06)	0.93 (0.78–1.11)
Co-negative for TGAb and TPOAb	10,193	5316	52.2	1	1

TGAb, thyroglobulin antibody; TPOAb, thyroid peroxidase antibody; LB, live birth; CLBR, cumulative live birth rate; OR, odds ratio

^a^ CLBR: cumulative live birth was defined as the delivery of one or more living infants in the first and subsequent frozen-thawed cycles. The cumulative live birth rate was calculated as the number of cumulative live births divided by the number of COS cycles

^*^ The multivariate model was adjusted for age, body mass index, cause of infertility, FT4, TSH, COS protocols, type of transfer (fresh vs. frozen), type of transferred embryo (cleavage-stage embryo vs. blastocyst), and fertilization method (IVF vs. ICSI).

Based on the titres of thyroid antibodies, women with isolated positivity for TgAb or TPOAb were further divided into high- and low-titre groups based on sample size. A total of 779 women with isolated positivity for TgAb were divided into 390 women with high titres (≥ 141.3 IU/mL) and 389 women with low titres (<141.3 IU/mL). No significant differences were observed between women with high and low TgAb titres. Among 580 women with isolated positivity for TPOAb, 290 were defined as high titres (≥ 217.1 IU/mL) and 290 women with low TPOAb titres (<217.1 IU/mL). No significant differences were observed between the two groups (Table 5).

**Table 5:** Subgroup analysis of cumulative live birth outcomes according to thyroid antibody titre

Group		No. of patients	No. of LB	CLBR (%) ^a^	OR (95% CI)	Adjusted OR (95%) ^*^
TGAb	High titres (≥ 141.3 IU/mL)	390	215	55.1	1	1
	Low titres (<141.3 IU/mL)	389	185	47.6	0.74 (0.56–0.98)	0.75 (0.56–1.01)
TPOAb	High titres (≥ 217.1 IU/mL)	290	144	49.7	1	1
	Low titres (<217.1 IU/mL)	290	142	49	0.97 (0.70–1.35)	0.98 (0.69–1.39)

TGAb, thyroglobulin antibody; TPOAb, thyroid peroxidase antibody; LB, live birth; CLBR, cumulative live birth rate; OR, odds ratio

^a^ CLBR: cumulative live birth was defined as the delivery of one or more living infants in the first and subsequent frozen-thawed cycles. The cumulative live birth rate was calculated as the number of cumulative live births divided by the number of COS cycles

^*^ The multivariate model was adjusted for age, body mass index, cause of infertility, FT4, TSH, COS protocols, type of transfer (fresh vs. frozen), type of transferred embryo (cleavage-stage embryo vs. blastocyst), and fertilization method (IVF vs. ICSI)

## Discussion

In our study, no significant difference was observed in the CLBR between the TAI and control groups, which suggests that women with TAI have a similar success rate in having a baby in a COS cycle to women without TAI. Our study firstly performed a subgroup analysis to investigate the impact of TAI on CLBR in women with different causes of infertility, which revealed a similar CLBR between women with TAI and women without TAI in different subgroups. We also demonstrated that different types and titres of thyroid antibodies did not influence CLBR.

Our study demonstrated similar CLBR between women with and without TAI, consistent with a previous study published in 2016 [10]. Studies on the association between TAI and CLBR are rare, many previous studies focused on investigating the association between TAI and assisted reproductive outcomes after the first embryo transfer cycle. Moreover, the conclusions varied among these different studies. A meta-analysis published in 2016 reviewed 12 cohort studies and revealed that TAI was associated with an increased risk of miscarriage and decreased chance of live birth [3]. In 2017, Aimee et al. published a secondary data analysis and reported that women with TAI had significantly higher miscarriage rates and lower live birth rates than those without TAI [11]. However, another meta-analysis published in 2020 that included 14 studies reported inconsistent results, revealing no significant difference in miscarriage and live birth rates between women with and without TAI [12]. A recent retrospective study published in 2023 also reported comparable miscarriage and live birth rates between women with and without TAI [7]. These controversial results may be attributed to differences in the study design, sample size, and population. However, an important confounding factor that should be considered in these studies is the instability of the first embryo transfer cycle. Compared with the live birth rate after the first transfer cycle, CLBR is a more stable outcome in assisted reproductive technology, which evaluates the possibility of having a baby from the first and all subsequent frozen-thawed transfer cycles, which more broadly reflects the quality of all embryos obtained from a complete COS cycle. Our results revealed that women with TAI have a similar possibility of having a baby as women without TAI in a complete COS cycle, which may challenge the value of screening for thyroid antibodies before COS treatment.

The cause of infertility is the most important confounding factor in the analysis of the association between the presence of TAI and assisted reproductive outcomes. Some studies reported that the presence of PCOS or endometriosis may increase the prevalence of TAI in women with infertility. In a meta-analysis of 13 studies evaluating 1,210 women with PCOS and 987 healthy controls, Romitti et al. reported a significantly higher prevalence of TAI in women with PCOS compared with healthy controls (OR 3.27, 95% CI 2.32–4.63) [13]. Another 2022 meta-analysis involving 7 case-control studies and 13 cross-sectional studies revealed patients with PCOS had a higher risk of developing thyroiditis (OR 2.28, 95%Cl 1.61–3.22) [14]. Petta et al. conducted a cross-sectional study between 148 women with surgically confirmed endometriosis and 158 controls, discovering a similar frequency of TAI between the two groups (OR 0.61; 95% Cl 0.34–1.11) [15]. However, another study that included 20 women with endometriosis and 100 controls reported a significantly higher prevalence of TAI in patients with endometriosis (OR 3.57, 95% Cl 1.09–11.8). This study also revealed no difference in the prevalence of TAI in couples with male-factor or tubal-factor infertility compared with controls [16]. The elevated prevalence of TAI suggests a potential interactive role between TAI and reproductive diseases. However, nearly all studies investigating the impact of TAI on assisted reproductive outcomes included populations with all causes of infertility and did not perform subgroup analyses due to the limited sample size. Our study is the first to perform a subgroup analysis based on different causes of infertility to analyse the impact of TAI on CLBR. No significant differences were observed between the TAI and control groups for all causes of infertility, except for infertility attributed to endometriosis. CLBR in women with endometriosis was significantly lower in the TAI group than in the control group; however, the difference was not significant after adjusting for potential confounders.

Whether the type and titre of thyroid antibodies affect CLBR remains uncertain. The distinction between TPOAb and TgAb has rarely been mentioned in previous studies. Many previous studies defined TAI as the presence of TPOAb, and populations with TgAb positivity were ignored. However, Unuane et al. discovered that compared with women without TAI, women who were co-positive for TgAb and TPOAb, along with women with isolated positive for TgAb had higher TSH levels. However, this association was not observed in women with isolated positive for TPOAb [17]. Another study revealed that women who were co-positive for TPOAb and TgAb had higher TSH levels than women with isolated positive for thyroid antibody, and that women with high titres of TPOAb or TgAb displayed an impaired thyroid response to human chorionic gonadotropin during pregnancy [18]. A recent meta-analysis also demonstrated an increase in TSH with isolated TPOAb or TgAb positivity, and the trend was amplified in individuals co-positive for both antibodies [19]. Studies on the impact of different types and titres of thyroid antibodies on pregnancy outcomes are limited. A previous study investigated the prevalence of different types and titres of thyroid antibodies in women with recurrent miscarriage and reported higher frequencies of TgAb alone and in association with TPOAb in women with recurrent miscarriage than in healthy women; however, the frequency of TPOAb alone was comparable. Moreover, higher titres of TgAb rather than TPOAb were also found in women with recurrent miscarriage [20]. Our previous study demonstrated that the type and titre of thyroid antibodies did not affect live birth rates after the first fresh embryo transfer cycle [9]. In this study, we first investigated the impact of different types and titres of thyroid antibodies on CLBR and compared with women without TAI. It was established that there was no significant difference in the CLBR among women co-positive for TPOAb and TgAb, and women with isolated positive for TPOAb or TgAb. Our study also revealed that the titres of thyroid antibodies did not affect CLBR in women with TAI.

Our study firstly performed a subgroup analysis to investigate the impact of TAI on CLBR in women with different causes of infertility. And in contrast to many previous studies, our study included TgAb to the diagnosis of TAI and analysed the combined impact of TPOAb and TgAb on CLBR in women with infertility. However, it is important to acknowledge that this study has several limitations. First, the data involved in our study were retrospectively collected, and some biases could not be avoided. Second, our study excluded patients with thyroid dysfunction before COS treatment, but did not investigate the use of levothyroxine before and during pregnancy due to its retrospective design. However, our previous randomized controlled trial has revealed that the use of levothyroxine did not affect IVF/ICSI outcomes [21].

## Conclusions

Among women with different causes of infertility, the presence of TAI did not affect the chance of having a baby in a complete COS cycle. Additionally, we conducted a subgroup analysis based on the causes of infertility and demonstrated the role of TAI in the CLBR of infertile women with different causes of infertility. The types and titres of thyroid antibodies were also considered in our study, and this is the first study to demonstrate no significant difference in CLBR between women with different types and titres of thyroid antibodies.

## Data Availability

The datasets used or analysed during the current study are available from the corresponding author on reasonable request.

## Abbreviations

TAI: Thyroid autoimmunity
PCOS: Polycystic ovarian syndrome
CLBR: Cumulative live birth rate
COS: Controlled ovarian stimulation
TgAb: Thyroglobulin antibody
TPOAb: Thyroid peroxidase antibody
IVF/ICSI: In vitro fertilization/intracytoplasmic sperm injection
GnRH: Gonadotropin-releasing hormone
BMI: Body mass index
AMH: Anti-Mullerian hormone
LH: Luteinizing hormone
TSH: Thyroid-stimulating hormone
FT4: Free thyroxine
SD: Standard deviation
ORs: Odds ratios
CIs: Confidence intervals
